# PERK promotes immunosuppressive M2 macrophage phenotype by metabolic reprogramming and epigenetic modifications through the PERK-ATF4-PSAT1 axis

**DOI:** 10.1097/IN9.0000000000000007

**Published:** 2022-07-29

**Authors:** Uday P Pratap, Ratna K Vadlamudi

**Affiliations:** 1 Department of Obstetrics and Gynecology, University of Texas Health Science Center at San Antonio, TX, 78229.; 2 CDP program, Mays Cancer Center, University of Texas Health Science Center at San Antonio, TX, 78229.; 3 Audie L. Murphy Division, South Texas Veterans Health Care System, San Antonio, TX 78229, USA.

**Keywords:** Endoplasmic reticulum (ER), ER stress (ERS), Protein kinase RNA-like ER kinase (PERK), M2 macrophage, Tumor associated macrophages (TAMs), myeloid cell-derived suppressor cells (MDSCs)

## Abstract

The endoplasmic reticulum (ER) is a specialized organelle that participates in multiple cellular functions including protein folding, maturation, trafficking, and degradation to maintain homeostasis. However, hostile conditions in the tumor microenvironment (TME) disturb ER homeostasis in cancer cells as well as immune cells. To overcome these conditions, cells activate ER stress response pathways which are shown to augment the suppressive phenotypes of immune cells; however, the molecular mechanisms underpinning this process remain elusive. Here, we discuss a recent study by Raines et al., that suggests the role of the helper T cell 2 (TH2) cytokine interleukin-4 (IL-4), and the TME in facilitating a protein kinase RNA-like ER kinase (PERK)-signaling cascade in macrophages, which promotes immunosuppressive M2 macrophage activation and proliferation. Further, the authors showed that PERK signaling promotes both mitochondrial respirations to fulfill cellular energy requirements and signaling through ATF-4 which regulate Phosphoserine aminotransferase 1 (PSAT1) activity to mediate the serine biosynthesis pathway. These results highlight a previously uncharacterized role for PERK in cellular metabolism and epigenetic modification in M2 macrophages, and thus offers a new therapeutic strategy for overcoming the immunosuppressive effects in the TME.

Cancer cells have a high growth rate; therefore, they have a sustained and enhanced demand for de novo protein synthesis, folding and maturation. Hostile environmental conditions such as hypoxia, oxidative stress, and chemotherapy jeopardize the fidelity of protein folding in the ER, resulting in ER stress (ERS) ^[1]^. In mammalian cells, the unfolded protein response (UPR) is initiated by three ER transmembrane proteins which act as a sensors of ER stress: inositol-requiring enzyme 1α (IRE1α), activating transcription factor 6 (ATF6), and PERK ^[2]^. However, persistent ER stress is associated with the adaptation of malignant cells in the TME and can disturb ER homeostasis in cancer and immune cells. An earlier study showed that the IRE1α arm of UPR signaling is crucial for macrophage polarization and dysregulation of the immune system in the TME ^[3]^. Nonetheless, function of other arms of UPR signaling in the regulation of macrophage metabolism and in the acquisition of immunosuppressive functions remain elusive.

Macrophages are key component of innate immune system and play a crucial role in maintaining tissue homeostasis and immunity ^[4]^. Macrophages can be further categorized as M0 (naive), M1 macrophages (proinflammatory) and M2 macrophages (immunosuppressive) ^[5]^. A recent study published in Nature Immunology discovered the PERK arm of UPR signaling augments the metabolic functions of macrophages and promotes an immunosuppressive M2 macrophage phenotype ^[6]^. In this study, the authors showed that Tumor associated macrophages (TAMs) isolated from lung carcinoma upregulates the PERK arm of the ER stress pathway. To gain mechanistic insights, the authors stimulated bone marrow-derived macrophages (BMDMs) isolated from mouse with Lipopolysaccharide (LPS) + Interferon-gamma (IFN-γ, M1 macrophage) and IL-4 (M2 macrophage) cytokines and performed RNA-seq analysis. Results indicated that macrophages stimulated with IL-4 (M2) has a high level of PERK in comparison to macrophages activated with LPS + IFN-γ (M1), which exhibited low levels of PERK activation. To further validate the role of PERK signaling in immunosuppressive M2 macrophages, the authors used myeloid-specific conditional knockout mice (*PERK*^cKO^) and showed that activation of PERK signaling promotes immunosuppressive M2 macrophages, however deletion of PERK attenuated this effect as well as restores the anti-tumor immune response ^[6]^.

Metabolic reprogramming of TAMs is very dynamic process and is influenced by the nutrient requirements of cancer cells, demands of tumor progression, and the course of chemotherapy. Interestingly, unbiased RNA-seq analysis of M2 macrophages from wild-type (WT) and *PERK*^cKO^ mice revealed that deletion of PERK downregulates the expression of several key genes responsible for lipid metabolism and mitochondrial respiration including lysosomal acid lipase (LIPA), peroxisome proliferator-activated receptor γ (PPAR-γ), and PPAR-γ coactivator-1β (PGC-1β), leading to a significant decreases in lipid intake, lipolysis, and energy intake in M2 macrophages ^[6]^. Another study showed that PERK signaling regulate mitochondrial autophagy by mitochondrial reprogramming and epigenetic changes in immune cells ^[7]^. To validate the PERK mediated regulation of mitochondria function, Raines et al., performed transmission electron microscopy in M2 macrophages isolated from WT and *PERK*^cKO^ mice. *PERK*^cKO^ M2 macrophages exhibited disorganized mitochondria cristae and lower mitochondrial numbers in comparison to WT M2 macrophages ^[6]^. Collectively, these results suggest that PERK signaling dysregulates M2 macrophage mitochondrial homeostasis.

Availability of serine, a non-essential amino acid is very essential to maintain mitochondrial homeostasis and function. Utilizing metabolomics approach, it was shown that serine derived lipids are very essential to support mitochondrial metabolism and that serine deprivation leads to mitochondrial defragmentation and alters mitochondrial fatty acid metabolism ^[8]^. Interestingly in this study, using metabolite profiling the authors found that the levels of intracellular 3-phosphoglycerate, serine and glycine were significantly elevated in IL-4 stimulated M2 macrophage compared to M1 macrophages. *PERK* deletion contributed to a decrease in serine levels as well as downregulated serine synthesis pathway gene *psat1*. Further, using myeloid cell conditional Psat1 knockout (*PSAT1*^cKO^) mice, Raines et al., demonstrated that serine metabolism is necessary for immunosuppressive M2 macrophages phenotype ^[6]^. Co-culture of phosphoserine aminotransferase 1 (*PSAT1*^cKO^) BMDMs with melanoma or lung cancer cell lines showed a significant diminution of the suppressive M2 macrophage phenotype suggesting that serine biosynthesis promotes the immunosuppressive M2 phenotype via the PERK-ATF4 pathway. Furthermore, the authors showed that PERK deletion resulted in alteration in α-KG production, and it was further dysregulated in *PSAT1*^cKO^ BMDMs. Using unbiased ChIP sequencing of PERK WT and *PSAT1*^cKO^ macrophages, the authors showed that α-KG plays critical role in metabolic adaptation and PERK–PSAT1 signaling facilitates an immunosuppressive M2 macrophage phenotype by regulating metabolic pathways and JMJD3 dependent histone demethylation ^[6]^.

Given the profound effect of PERK signaling on M2 macrophage metabolic reprogramming and immunosuppressive function, few other studies examined whether inhibition of PERK signaling could elicit anti-tumor immune responses ^[9,10]^. The results from these studies showed that UPR mediated PERK activation led to the transformation of myeloid cells into tumor-associated myeloid-derived suppressor cells. Further, PERK deletion reprogramed tumor-myeloid cell-derived suppressor cells (MDSCs) into immunostimulatory cells, augmented CD8^+^ T cell response and showed a synergistic response in combination with immunotherapy; suggesting PERK inhibition is a promising therapeutic strategy in cancer. Similarly, in this study Raines and colleagues examined the effect of the PERK inhibitor (GSK2656157) and PHGDH inhibitor (NCT-503) on B16-F10 melanoma xenografts and observed a significant reduction in tumor growth and number of immunosuppressive TAMs. Furthermore, treatment with the PERK inhibitor induced an anti-tumor response by increasing both CD8^+^ and CD4^+^ T cell populations and the overall survival of melanoma bearing mice. Follow-up experiments revealed that combined treatment of the PERK inhibitor with immune checkpoint blockage/anti Programmed Cell Death Protein 1 (PD-1) immunotherapy enhance the anti-tumor efficacy in tumor bearing mice. These study results suggest that the PERK signaling inhibitor could be used to transform the immunosuppressive M2 macrophage phenotype to ani-tumor phenotype ^[6]^.

In summary, Raines et al. study uncovered PERK directed rewiring of metabolic circuits and epigenetic changes through the PERK-ATF4-PSAT1 axis as a novel mechanistic pathway in the modulation of TME. These exciting findings opened new avenues in the field of translational immunometabolism (Figure 1). Further, this study provided new directions in exploring the interplay between metabolomics and epigenetic changes, which will have wide implications to combat cancer and other metabolic and inflammatory diseases. Therefore, targeting PERK signaling could be used to modulate anti-tumor immune response by regulating immunosuppressive M2 macrophage metabolism. This strategy could also be used to target additional key proteins involved in metabolic reprogramming of the immune system in cancer. In addition, such therapies can also be extended for treating inflammatory diseases. Imperatively, future studies are needed to fill the large knowledge gap in the translation of pre-clinical study findings to clinical trials.

## Figures and Tables

**Figure 1.**
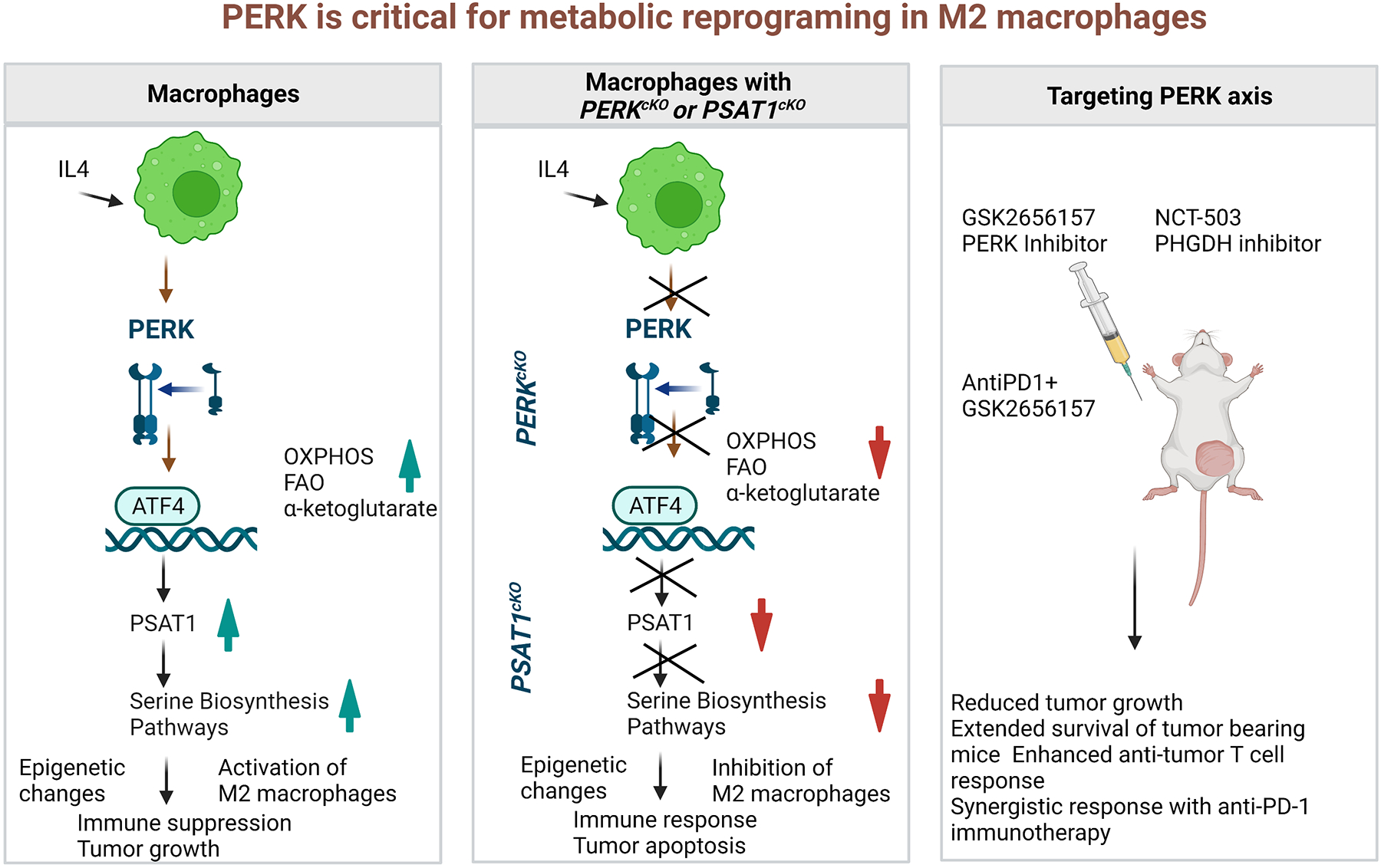
A schematic presentation of critical role of PERK in metabolic reprogramming of macrophages via epigenetic changes. (**a**) IL-4 stimulation to WT macrophages results in activation of immunosuppressive M2 macrophage via serine biosynthesis pathway (SBP). (**b**) IL-4 stimulation to *PERK*^cKO^ macrophages or *PSAT1*^cKO^ macrophages results in alteration in SBP, which is responsible to drive the immunosuppressive epigenetic modifications in M2 macrophages. (**c**) Treatment of mice with GSK2656157 (PERK inhibitor) or NCT-503 (PHGDH inhibitor) reduce tumor burden as a monotherapy and combination therapy of AntiPD1 + GSK2656157 potentiate the antitumor response.
